# Aminopeptidase N (APN)/CD13 inhibitor, Ubenimex, enhances radiation sensitivity in human cervical cancer

**DOI:** 10.1186/1471-2407-8-74

**Published:** 2008-03-19

**Authors:** Hirohisa Tsukamoto, Kiyosumi Shibata, Hiroaki Kajiyama, Mikio Terauchi, Akihiro Nawa, Fumitaka Kikkawa

**Affiliations:** 1 Department of Obstetrics and Gynecology, Nagoya University Graduate School of Medicine, Nagoya, Japan

## Abstract

**Background:**

Radiotherapy can be used to treat all stages of cervical cancer. For improving local control via radiotherapy, it is important to use additional antitumor agents. Aminopeptidase N (APN)/CD13, a 150-kDa metalloproteinase, is a multifunctional cell surface aminopeptidase with ubiquitous expression. Recent studies have suggested that APN/CD13 plays an important role in tumor progression in several human malignancies.

**Methods:**

We investigated whether the suppression of APN/CD13 using Ubenimex, an inhibitor of APN/CD13 activity, may affect tumor radiosensitivity in cervical cancer cells both *in vitro *and *in vivo*. Cell surface APN/CD13 activity in HeLa cells was calculated using alanine-p-nitroanilido as a substrate. For colony formation assays, single-dose radiation and/or Ubenimex were administered to each dish of HeLa cells, and these dishes were cultured for 14 days. Molecular changes of apoptosis were determined by Western blot. Apoptosis was evaluated by Annexin-V PI staining (flow cytometry analysis) and the Tunel method. Moreover, we investigated the effect of combining Ubenimex and low-dose radiation on tumor growth using nude mice.

**Results:**

We demonstrated that Ubenimex enhanced the effectiveness of radiotherapy, acting as a radiosensitizer both *in vitro *and *in vivo*. In colony formation assays, a significant decline in clonogenic survival was observed in Ubenimex-treated cells. Mice treated with a combination of radiation and Ubenimex showed a significant prolongation of the tumor-doubling time compared with the control, Ubenimex, or radiation-alone groups. We also showed that ubenimex enhanced radiation-induced apoptosis *in vitro *and *in vivo*.

**Conclusion:**

Although further studies are needed, this report suggests that Ubeniemx acts as a radiosensitizer in cervical cancer treatment, and that the inhibition of APN/CD13 activity may represent a new approach for improving the therapeutic efficacy of radiotherapy for uterine cervical cancer.

## Background

Uterine cervical cancer is still the second most common cancer in women worldwide, despite the existence of effective screening methods [1]. Radiation therapy can be used to treat all stages of cervical cancer. Many cervical cancers are very radiosensitive, and radiotherapy gives good results; however, residual disease causes clinical relapse. Thus, for improving the local control of cervical cancer, the combination of radiation with additional anti-tumor agents is warranted. Against this background, concurrent chemoradiation with a cisplatin-based regimen has recently been used to treat advanced cervical cancer [2-6]. However, the treatment causes strong side effects such as digestive symptoms (vomiting and diarrhea) and bone marrow suppression. Thus, drugs with fewer side effects and a superior effect in combination are desired.

Aminopeptidase N (APN)/CD13 is a zinc-binding type 2 transmembrane ectopeptidase of 150 kDa that forms a noncovalently bound homodimer on the cellular membrane. Although APN/CD13 was first described as a marker for hematopoietic cells of myeloid origin, expression has been reported on non-hematopoietic cells and tissues, such as fibroblasts, brain cells, and epithelial cells of the liver, kidney, and intestine. High expression levels of APN/CD13 have been detected in various solid tumors [7-9]. The expression level of APN/CD13 was found to correlate with increased malignant behavior in prostate, colon, and non-small cell lung cancer [10-12]. Ubenimex, a CD13/aminopeptidase N (APN) inhibitor, has been shown to be cytotoxic to tumor cell lines *in vitro*, and it dose-dependently inhibits the growth of lung cancer and leukemic cell lines [13,14]. However, the detailed antitumor mechanism of Ubenimex remains unclear.

In the current study, to improve the therapeutic efficacy of radiotherapy for cervical cancer, we investigated the potential of Ubenimex to act as a radiosensitizer. Initially, *in vitro *studies showed that Ubenimex potentiated radiation-induced cell killing in a clonogenic assay. This increased cell killing was correlated with the up-regulation of caspase-3 and PARP cleavage, suggesting the activation of apoptotic signaling pathways.*In vivo*, the combination of Ubenimex with cervical cancer irradiation led to an enhancement of tumor growth inhibition and increased cancer tissue apoptosis. Our study demonstrated the potential for the use of Ubenimex to improve the effectiveness of clinical strategies in cervical cancer radiotherapy.

## Methods

### Cell culture

The human uterine cervical cancer cell line (HeLa) was used. HeLa cells were kindly provided by Dr. Ruey Min Lee (Huntsman Cancer Institute, Salt Lake, UT, USA). Cells were maintained in RPMI-1640 (Sigma, St. Louis, MO, USA) supplemented with 10% FCS and penicillin-streptomycin. These cells were incubated at 37°C in a humidified atmosphere of 5% CO_2_.

### Irradiation

The cells were irradiated at doses between 2 and 14 Gy at room temperature using an MBR-1505R2 X-ray generator (Hitachi Medical Co.) delivering 0.8 Gy/min.

### Enzyme activity assay

Cell surface APN/CD13 activity in HeLa cells was detected spectrophotometrically, as reported by Amoscato *et al*. [15]. After incubating 4 × 10^5 ^cells in a 60 × 15 mm culture dish for 24 h at 37°C, each dish was irradiated with doses of 16 Gy. Twelve hours following, aspirating off the medium and washing with PBS, 1 mM alanine-p-nitroanilido (Peptide Institute, Inc.) was added to each dish as a substrate. Each dish was then incubated at 37°C for 60 min. The supernatant was subsequently collected, and 200 μl of the solution was added to 96-well microtiter plates. APN/CD13 enzyme activity was measured by a microplate reader (Tecan; λ_exc _of 405 nm and λ_em _495 nm) (Labsystems, Multiskan Bichromatic, Helsinki, Finland).

### Clonogenic survival

For colony formation assays, cells (from 100 to 1 × 10^5 ^cells) in a 60 × 15 mm culture dish were seeded in triplicate according to treatment conditions and cell lines. After allowing cells to attach to the dishes, a single dose of radiation and/or Ubenimex was given. Cells were always pretreated with Ubenimex for 24 h before radiation when the two treatments were combined. Cells were cultured for up to 14 days. Colonies were then fixed, stained with crystal violet, and counted. The surviving fraction was estimated as follows: (number of colonies formed)/(number of cells seeded) × [plating efficiency for the no-treatment or Ubenimex group (control group)].

### Western blot analysis

Cells were grown to 70–80% subconfluence and treated with lysis buffer containing 1% Triton ×-100 in PBS and protease inhibitor mixture tablets (Roche, Barcelona, Spain). Ten μg of total cell lysate were electrophoresed on a 10% SDS-polyacrylamide gel and transferred electrophoretically to Immobilon membranes (Millipore, Bedford, MA, USA). After adding blocking solution (5% nonfat dry milk/0.1% Tween-20/PBS), the membranes were incubated overnight with a recommended dilution of primary antibodies. We used anti-Bcl-xL (Cell Signaling BD, 2760), anti-Bcl-2 (Cell Signaling BD, 2872), anti-caspase-3 (Santa Cruz, sc-7272), anti-cleaved caspase-3 (Cell Signaling, 7190), anti-PARP (Cell Signaling, 9542), anti-cleaved PARP (Cell Signaling, 9541), and anti-β-actin antibodies (Sigma, AC-74). The primary antibodies were washed in 0.05% Tween-20/PBS and then incubated with horseradish peroxidase-conjugated secondary antibody. Proteins were visualized using enhanced chemiluminescence reagent (Amersham Pharmacia Biotech) followed by exposure to X-ray film.

### Flow cytometric analysis for apoptosis

To quantify the apoptotic death of HeLa cells, annexin V and PI (propidium iodide) staining was performed, followed by flow cytometry. Cells were plated at 4 × 10^5 ^cells in a 60 × 15 mm culture dish for 24 h, and treated with 16 Gy irradiation. After treatment for 72 h, attached cells were collected by brief trypsinization, washed twice with PBS, and then subjected to annexin V and PI staining using a MEBCYTO apoptosis kit (MBL). After staining, quantitative analysis for apoptosis was performed by flow cytometry.

### Tumor model and treatment conditions

Female nude mice (BALB/c) at 6 weeks were provided from Chubu Kagaku (Nagoya, Japan). HeLa cells (1 × 10^7 ^cells/0.5 ml of medium/mouse) were injected into the left flank of animals. Before initiating the study, 20 mice were assigned to four groups.

Each group contained an equal number of large and intermediate-sized tumors, and mice were stratified into groups so that the mean tumor volume in each group was comparable.

(a) Control group (no radiation, sterile PBS injections),

(b) Radiation alone group (locally radiated at a dose of 4 Gy, sterile PBS injections),

(c) Ubenimex alone group (treatment with Ubenimex for 7 days at 10 mg/kg/day), and

(d) Combination treatment group (concomitant radiation plus Ubenimex for 7 days at 10 mg/kg/day scheduling: started 24 hours before 4 Gy radiation).

For radiation treatment, mice were immobilized in a customized harness facilitating exposure of the left flank, whereas the remainder of the body was shielded by lead.

4 Gy radiation was delivered locally in one fraction on day 7 using an MBR-1505R2 X-ray generator (Hitachi Medical Co.) delivering 0.8 Gy/min.

Intratumoral injections of Ubenimex began 24 h before radiation and were continued daily for 7 days. The tumor volume was estimated from two-dimensional tumor measurements by the formula: V = length (mm) × width^2 ^(mm^2^)/2.

The unpaired Student's *t*-test was used to determine the significance of the relative tumor volumes and for comparisons between groups.

### TUNEL assay

The TUNEL assay was carried out using MEBSTAIN Apoptosis KitII(MBL CO., LTD., Nagoya, Japan). DNA fragmentation was detected by this assay. Paraffin-embedded sections (4 μm thick) of mice were cut and mounted on precoated glass slides. Sections were deparaffinized prior to digestion with 20 μg/mL proteinase K for 15 min at room temperature. The slides were washed 4 times in distilled water for 2 min and covered with 2% hydrogen peroxide in phosphate-buffered saline (PBS) for 5 min at room temperature to inactivate endogenous peroxidase. The slides were rinsed with PBS twice and immersed in TdT-containing buffer (30 mmol/L Triazma base, pH 7.2, 140 mmol/L sodium cacodylate, and 1 mmol/L cobalt chloride) for 15 minutes to prepare digoxigenin-binding sites. An antidigoxigenin antibody fragment carried a conjugated reporter enzyme (peroxidase) to the reaction sites, and then localized peroxidase generated an intense signal from the chromogenic substrate diaminobenzidine. The counterstain was made by methyl green. Apoptosis was defined as the number of stained positive cells under a 200 × fluorescence microscope.

### Statistical analysis

Data were obtained from three individual experiments performed in triplicate. Statistical analysis was performed using the unpaired Student's *t*-test. Differences were considered significant at *p *< 0.05.

## Results

### Radiation increased APN/CD13 enzyme activity in uterine cervical cancer cell line

**Figure 1:**
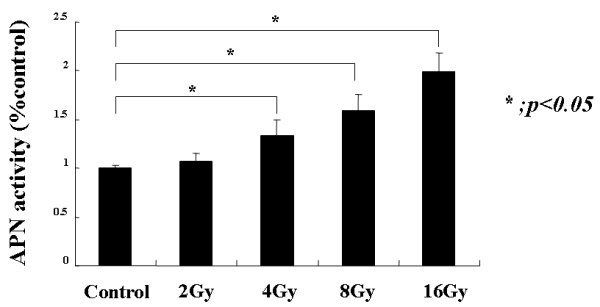
**APN/CD13 enzyme activity in HeLa cells with or without (control) of radiation (16 Gy).** Enzyme activity was determined by quantitating the enzymatic cleavage of the synthetic substrate alanine-p-nitroanilido. After 1 hour incubation, APN/CD13 activity is depicted as about two fold increase in HeLa cells after radiation. Radiation increased APN/CD13 enzyme activity. Errors bar represent the standard error of the mean.

We first investigated whether radiation increased the cell surface APN/CD13 activity of HeLa cells. APN/CD13 enzyme activity in HeLa cells with or without of irradiation (16 Gy) was investigated. Enzyme activity was determined by quantitating the enzymatic cleavage of the synthetic substrate alanine-p-nitroanilido. After a1-h incubation, APN/CD13 activity is depicted as about two fold increase in HeLa cells after radiation (Fig. 1).

### Increased HeLa cell killing by Ubenimex and radiation

**Figure 2:**
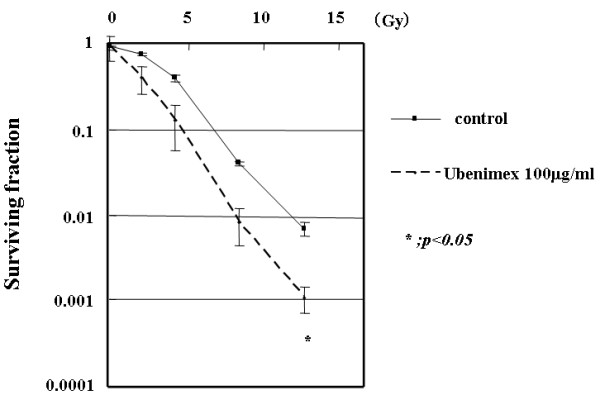
**Clonogenic survival of HeLa cells with or without 100 μg/ml Ubenimex.** Colonies were counted 14 days after radiation. Errors bar represent the standard error of the mean.

As radiation increased APN/CD13 activity, we investigated the potential of Ubenimex to act as a radiosensitizer. HeLa cells were pretreated with 100 μg/ml Ubenimex for 24 h, irradiated with 2–14 Gy, and then plated in a 14-day clonogenic assay. The survival curves obtained for radiation alone and Ubenimex-treated cells in combination with irradiation are shown in Fig. 2. A significant decline in clonogenic survival was observed in Ubenimex treated cells in combination with radiation of 14 Gy (*p *< 0.05).

### Ubenimex enhanced irradiation-induced apoptosis

**Figure 3:**
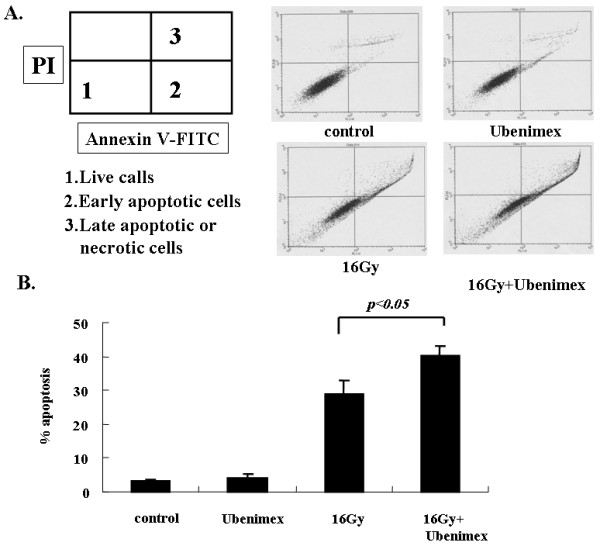
**(A)Annexin V and PI (propidium iodide) staining was performed, followed by flow cytometry.** (B) The percentage of apoptotic cells was counted (Fig. 3A, areas 2 and 3). Almost 3–4% apoptosis was found in the control and Ubenimex groups. In the radiation and combined-treatment groups there was a high percentage of apoptosis. Combined treatment with Ubenimex and radiation resulted in a higher percentage of apoptosis than in the radiation alone group (*p *< 0.05). Errors bar represent the standard error of the mean.

To investigate whether the induction of apoptosis plays a role in Ubenimex-induced radiosensitization, annexin V and PI (propidium iodide) staining by flow cytometry was performed (Fig. 3A and 3B). The level of apoptosis was analyzed at 72 h after 16 Gy radiation, because, at this time point, the highest level of apoptosis was noted. The percentage of apoptotic cells was counted (Fig. 3A, areas 2 and 3). Almost 3–4% apoptosis was found in the control and Ubeninex-alone groups. However, in the 16 Gy radiation-alone and combined-treatment groups, there was a high percentage of apoptosis. Further, combined treatment with Ubenimex and radiation resulted in a higher percentage of apoptosis than in the 16 Gy radiation-alone group. A significant difference was observed between only radiation and combined treatment with Ubenimex and radiation (*p *< 0.05).

### Activation of apoptotic pathway by combination of Ubenimex and radiation

**Figure 4:**
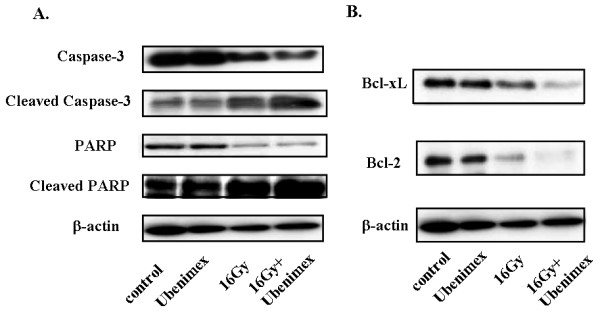
**Modulation of apoptotic molecules by Ubenimex and radiation.** HeLa cells were pretreated with 100 μg/ml Ubenimex for 24 h, followed by 16 Gy radiation. At 72 h after radiation treatment, whole-cell protein extracts were prepare and subjected to Western blot analysis of Bcl-xL, Bcl-2, caspase-3, cleaved caspase-3, PARP, and cleaved PARP. The β-actin protein level served as a protein loading control. The combination of Ubenimex and radiation cleaved caspase-3 and PARP, leading to apoptosis (A). Anti-apoptotic molecules, Bcl-xlL and Bcl-2, were down-regulated by Ubenimex and radiation (B).

We investigated the effect of Ubenimex on the modulation of molecules involved in the apoptotic pathway. The expression of molecules related to apoptosis was determined by Western blot analysis. As shown in Fig. 4A, the combination of Ubenimex and radiation enhanced the expression of cleaved caspase-3 and PARP, leading to apoptosis. Also, anti-apoptotic molecules, Bcl-xL and Bcl-2, were down-regulated by Ubenimex and radiation (Fig. 4B). These results showed that the combination of Ubenimex and radiation activated the apoptotic pathway more strongly than the other treatments.

### Effect of combination of Ubenimex and low-dose radiation on tumor growth *in vivo*

**Figure 5:**
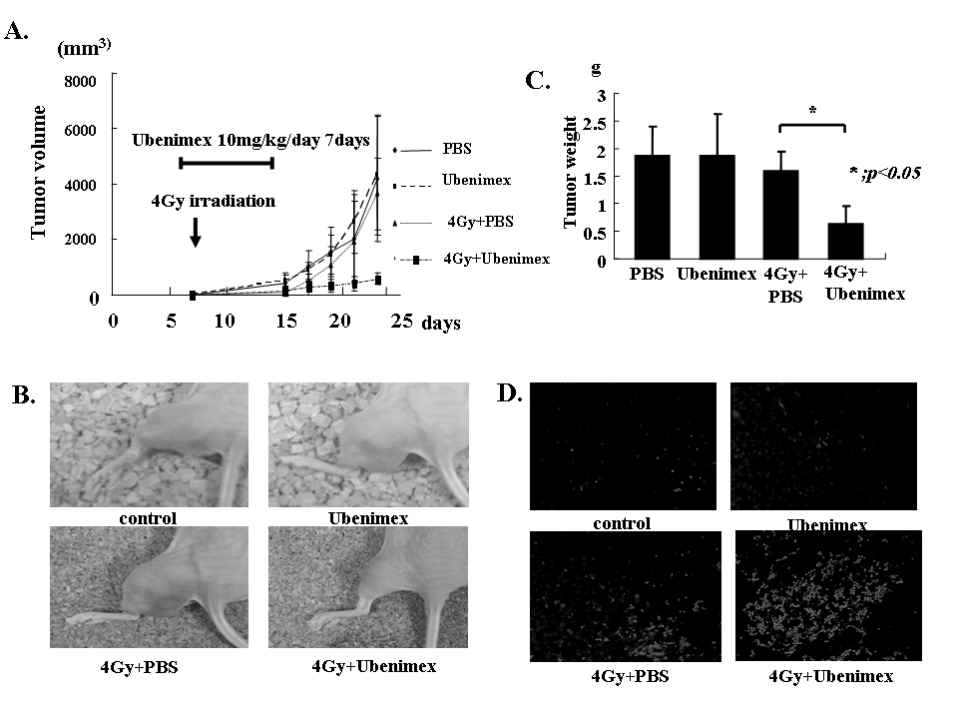
**(A) Effect of Ubenimex alone or combined with radiation on s.c.** HeLa tumor volumes (mm^3^) over time (days). Mice were treated with PBS, bestatin, 4 Gy+PBS, and 4 Gy+bestatin, and tumor volumes were monitored over time. Mice were sacrificed on 23^rd ^day. (B) Photographs of tumors on the 23^rd ^day. (C) Tumor weight on the 23^rd ^day. Optimal growth inhibition was observed in the 4 Gy+bestatin group compared with the other groups. (D)TUNEL assay of mouse tumor sections. This method allows the direct detection of DNA fragmentation. The bright cells are apoptotic cells. More apoptotic cells were observed in the combined-treatment group than in the other groups. Errors bar represent the standard error of the mean.

The effect of combined treatment with Ubenimex and radiation on the growth of HeLa tumors was tested in four separate experiments. As can be seen in Fig. 5A, mice that received combined treatment of 4 Gy radiation and Ubenimex exhibited much smaller tumors compared with mice that received no treatment or were treated with only radiation or Ubenimex. In terms of the tumor volume doubling time, the Ubenimex-alone group showed no difference compared with the no-treatment group (2.4 versus 2.5 days, respectively). In contrast, the combined-treatment group showed a significantly longer doubling time than that of the radiation alone group (17.8 days versus 11.5 days, respectively). As shown in Fig. 5B, to the eye, tumor appearance in the combined-treatment group was different from the tumors of the other groups. Regarding tumor volume, optimal growth inhibition was observed in the combination treatment group compared with the radiation alone group (*p *< 0.05; combined-treatment group versus ionizing irradiation) (Fig. 5C). Finally, we used the TUNEL method to visualize DNA fragmentation at the single cell level. As shown in Fig. 5D, more apoptotic cells were observed in the tumor xenografts of the combined-treatment group than in the other groups.

## Discussion

In the current study, we first focused on Ubenimex as a drug used in combination with radiation in cervical cancer treatment. The combined antitumor activity of radiation and Ubenimex was tested both *in vitro *and *in vivo*. In our animal model, treatment with Ubenimex alone did not influence tumor growth in nude mice inoculated s.c. with HeLa cells. However, combined treatment with Ubenimex and radiation resulted in a significantly higher response rate compared with radiation alone. These results are consistent with *in vitro *data shown in Figs. 2 and 3. In this study, the combined treatment was markedly effective in vivo, but not in vitro. This is presumably because we used relatively large numbers of cells in in-vitro assays. In addition, our apoptosis assay results include those of assays for late apoptosis and necrosis, and so further studies using low-dose radiation are needed. The mechanism by which APN/CD13 activity functions in the regulation of radiosensitivity remains to be clarified. APN/CD13 has been suggested to be involved in the degradation of neuropeptides, cytokines, and immunomodulatory peptides, as well as angiotensins [16,17]. Thus, it is possible that APN/CD13 activity may contribute to radioresistance through proteolytically modifying peptides involved in anti-apoptotic signaling. Ubenimex inhibits APN/CD13, aminopeptidase B, and leucine aminopeptidase of mammalian cells [18]. An antigrowth or antimigratory effect is frequently observed in studies using Ubenimex or anticatalytic antibodies for APN/CD13 in several malignancies [19-23]. Our results show that Ubenimex acted as a radiosensitizer to enhance radiation-induced apoptosis in cervical cancer cells. This finding suggests that the concomitant use of Ubenimex during radiotherapy could be a potential therapeutic approach to improve the efficacy of radiotherapy for uterine cervical cancer.

The mechanism involved in the combined treatment of Ubenimex and radiation remains unknown; the two agents achieve cytotoxicity through different means. Previous studies have shown that Ubenimex directly induces the apoptosis of human leukemia and non-small-lung cancer cells [13,14]. Furthermore, previous studies have shown that Ubenimex enhances apoptosis or increases sensitivity to antineoplastic agents [24-27]. Consistent with these findings, the present data suggested that Ubenimex enhanced the radiosensitivity of cervical cancer. In this study, we speculated that Ubenimex would induce apoptosis by inhibiting the radiation-induced activity of APN/CD13. However, no significant apoptosis induction was observed after the expression of APN/CD13 was knocked out by siRNA (data not shown). One of possible explanation for this observation is that Ubenimex may inhibit other aminopeptidase activities, and thereby influence the cell cycle or apoptotic signals, since it is not completely specific for APN/CD13. Santos et al. demonstrated that APN/CD13 was directly involved in signal transduction pathways, including the phosphorylation of mitogen-activated protein kinases, such as ERK1/2, JNK, and p38, in monocytes [28]. Although the detailed mechanisms are still under investigation, APN/CD13 may be involved in signaling cascades regulating cell survival, protection from apoptosis, and radiosensitivity via aminopeptidase-dependent and independent mechanisms.

This study may be controversial in that 50 times the clinical dose of Ubenimex was administered. Ubenimex is an immunomodulator with few side effects, but may cause mild nausea, vomiting, and liver dysfunction. In addition, further studies are needed to investigate the methods of Ubenimex administration. In conclusion, the data obtained here suggested that APN/CD13 activity was induced with radiation, and the inhibition of APN/CD13 may lead to the restoration of radiosensitivity in cervical cancer cells. Although there are always limitations when one attempts to extrapolate from mice to humans, our study suggests that combination with ubenimex may provide additional benefits regarding the radiosensitivity of cervical cancer.

## Conclusion

This work demonstrated that inhibition of APN/CD13 activity may represent a new approach for improving the therapeutic efficacy of radiotherapy for uterine cervical cancer.

## Competing interests

The author(s) declare that they have no competing interests.

## Authors' contributions

HT participated in study design, performed cell culture, enzyme activity assay, colony formation assays, and flow cytometry analysis. KS participated in study design, performed western blot analysis, drafted and revised the manuscript. HK participated in study design, performed TUNEL assay. MT performed animal experiment and statistical analysis. AN participated in study design, performed animal experiment. FK participated in study design and coordination, supervision of experimental conduct and analysis, drafting and revision of the manuscript, and approved the final version. All authors have read and approved the final manuscript.

## Pre-publication history

The pre-publication history for this paper can be accessed here:


